# The Gene Ontology's Reference Genome Project: A Unified Framework for Functional Annotation across Species

**DOI:** 10.1371/journal.pcbi.1000431

**Published:** 2009-07-03

**Authors:** The Reference Genome Group of the Gene Ontology Consortium

**Affiliations:** University of California San Diego, United States of America

## Abstract

The Gene Ontology (GO) is a collaborative effort that provides structured vocabularies for annotating the molecular function, biological role, and cellular location of gene products in a highly systematic way and in a species-neutral manner with the aim of unifying the representation of gene function across different organisms. Each contributing member of the GO Consortium independently associates GO terms to gene products from the organism(s) they are annotating. Here we introduce the Reference Genome project, which brings together those independent efforts into a unified framework based on the evolutionary relationships between genes in these different organisms. The Reference Genome project has two primary goals: to increase the depth and breadth of annotations for genes in each of the organisms in the project, and to create data sets and tools that enable other genome annotation efforts to infer GO annotations for homologous genes in their organisms. In addition, the project has several important incidental benefits, such as increasing annotation consistency across genome databases, and providing important improvements to the GO's logical structure and biological content.

## Author Summary

Biological research is increasingly dependent on the availability of well-structured representations of biological data with detailed, accurate descriptions provided by the curators of the data repositories. The Reference Genome project's goal is to provide comprehensive functional annotation for the genomes of human as well as eleven organisms that are important models in biomedical research. To achieve this, we have developed an approach that superposes experimentally-based annotations onto the leaves of phylogenetic trees and then we manually annotate the function of the common ancestors, predicated on the assumption that the ancestors possessed the experimentally determined functions that are held in common at these leaves, and that these functions are likely to be conserved in all other descendents of each family.

## Introduction

### Background

The functional annotation of gene products, both proteins and RNAs, is a major endeavor that requires a judicious mix of manual analysis and computational tools. The manual aspect of this annotation task is carried out by curators, from the Latin *cure*: to look after and preserve. A curator in this context is a Ph.D. trained professional life scientist whose task is to meaningfully integrate published, and in some cases unpublished, biological data into a database [1],[2].

The GO was developed within the community of the Model Organism Databases (MODs), whose goal is to annotate the genomes of organisms having important impact on biomedical research [3],[4]. The GO consists of over 26,000 terms arranged in three “branches”: molecular function, biological process, and cellular component. Terms are related to each other by well-defined relationships, particularly by a subsumption relationship (is_a), a partitive relationship (part_of) and relationships which denote biological regulation (regulates). GO is one of the most widely used tools for functional annotation, particularly in the analysis of data from high throughput experiments. GO terms are manually associated with gene products by curators using two general methods: extracting annotations based on published experimental data; and inferring annotations based on homology with related gene products for which experimental data is available. Automated methods that are based on either sequence similarity or domain composition are also used to make annotations without curator intervention. These different methods of assigning GO terms to gene products are distinguished by the use of different GO evidence codes [5]. The comprehensive annotation of a genome entails assigning functions to all gene products, including those that have not yet been experimentally characterized.

### Motivation

The annotations based on experimental data provide a solid, dependable substrate for downstream analyses to infer the functions of related gene products. High-quality manual annotation by experts is an absolute prerequisite for seeding this system and, other than the major MOD projects and large sequence databse projects (such as UniProt and Reactome), very few research communities have the resources or trained GO curators to perform this labor-intensive task. Therefore, the functional annotation of non-manually curated genomes typically relies on automated methods that provide the core information for the transfer of annotations from related genes for which experimentally supported annotations *are* available.

The GO Reference Genome project is committed to providing comprehensive GO annotations for the human genome, as well as that of eleven important model organisms: *Arabidopsis thaliana*, *Caenorhabditis elegans*, *Danio rerio*, *Dictyostelium discoideum*, *Drosophila melanogaster*, *Escherichia coli*, *Gallus gallus*, *Mus musculus*, *Rattus norvegicus*, *Saccharomyces cerevisiae*, and *Schizosaccharomyces pombe*. Collectively those twelve species are referred to as the “GO Reference Genomes”. Each model organism has its own advantages for studying different aspects of gene function, ranging from basic metabolic reactions to cellular processes, development, physiology, behavior, and disease. The organisms selected to provide this gold-standard reference set have the following characteristics: they represent a wide range of the phylogenetic spectrum; they are the basis of a significant body of scientific literature; a reasonably sized community of researchers study the organism; and the organism is an important experimental system for the study of human disease, or for economically important activities such as agriculture. Importantly, all of these organisms are supported by an established database that includes GO curators who have the expertise to annotate gene products in these genomes according to shared, rigorous standards set by the groups participating in the Reference Genome project (see below).

Although the development of the GO has been a collaborative effort since its inception, each participating group has previously worked independently in assigning GO annotations. Thus, prior to this project, specific protocols for annotation varied greatly between the different databases. Variation in annotation results from different curator decisions as to which data is appropriate to annotate and which GO terms to employ. [6],[7]. Other discrepancies in annotations come from the use of different methods to perform “automated annotations” (primarily based on comparisons of homologous genes) by each of the different groups. Those two factors contribute to the inconsistencies observed among propagated annotations [8]–[11]. To address this issue, it was decided that the groups would simultaneously curate a number of homologous genes to provide an opportunity for improving the accuracy and consistency of the annotations made by the different groups. This strategy has the additional benefit of improving the ontology, since several curators working simultaneously with particular nodes of the GO structure can collaboratively identify omissions, ambiguities or logical inconsistencies in the GO and work towards their resolution with the ontology editors.

### Impact

We expect these reference annotations to have two important applications. First, they will increase the quality of the annotations provided by the GO Consortium, with a focus on providing precise annotations for each gene and the broadest possible coverage of each genome. Second, the gold-standard annotation set will greatly accelerate the annotation of new genomes where extensive experimental data on gene function or the resources and expertise to perform the annotations are unavailable.

## Methods

There are two different aspects of comprehensive annotation: “breadth” and “depth”. *Depth* refers to the amount of information about each gene that has been captured. For maximal depth, annotations should be as precise as possible; ideally, all experimentally determined information (primarily from the biomedical literature) about the gene products from each of these organisms should be curated to the deepest level in the gene ontology graph. *Breadth* refers to the coverage of the genome, that is, the percentage of genes annotated. For maximal breadth, the annotations would ideally cover every gene product in a genome. From a production standpoint, these dual aspects imply a dependency, that is, we must carry out curation in two passes: first literature-based annotation of to capture all information based on experiments, followed by the inference of annotations to the homologous gene products that have not yet been experimentally characterized. Finally, it is important to distinguish genes for which the function is actually unknown from genes that simply have not yet been annotated. To this end, reviewed proteins for which there is no experimental data and that do not share significant homology with experimentally characterized proteins are annotated to the root term of each ontology: biological process (GO:0008150), molecular function (GO:0003674), and cellular component (GO:0005575).

This procedure maximizes both depth and breadth of annotation across all of the curated genomes. We refer to the annotations as ‘comprehensive’ rather than ‘complete’ because it is not always feasible to completely annotate every published paper for every gene with our resources. For genes with a large body of literature, the comprehensiveness of annotations is assessed by curators based on a recent review or text-mining applications.

### Concurrent annotation approach

One major advantage of annotating several genomes concurrently is the ability to carry out parallel annotations on homologous genes. Annotating several genes in a single step improves annotation efficiency. Moreover, it improves breadth of annotations by allowing easy access to known function of related genes. Finally, concurrent annotation of gene families across different databases promotes annotation consistency.

#### Generating sets of homologous genes

The organisms represented in the GO Reference Genome project span well over 1 billion years of evolutionary divergence. The premise that underpins the comparative genomics approach is that homologous genes descended from a common ancestor often have related functions. This is not, of course, to deny that genes will diverge in function, but it is generally true that at least some aspects of function are conserved (particularly if there has been relatively little sequence divergence, which can be established using the sequence data alone). For our purposes, a critical first step is the establishment of a standard approach to determining sets of homologous genes. Ideally, the evolutionary history of each gene in all organisms would be analyzed and stored in a single resource that could be used as the definitive reference for gene family relationships and homologous gene sets. However, generating this resource is a non-trivial problem, both theoretically, as just described, as well as practically. At present no single resource offers a fully satisfactory solution. Different resources exist that provide different results in terms of specificity and coverage and have different strengths and weaknesses [12]–[14].

One central confounding problem has been the lack of a “gold standard” protein set that would be used by all databases and homology prediction tools. Because the different homology prediction tools do not use the same protein sets as inputs their results cannot be meaningfully compared. Moreover, the protein sets that are being annotated by the GO Consortium members may, and often do, differ from those used by the different homology prediction programs. The GO Consortium is now providing an index of protein sequence accession identifiers for each organism to groups who compute homology sets (see “*Data availability*” below). The P-POD [15] and PANTHER [16],[17] databases are already using these sets, with PANTHER computing phylogenetic trees and P-POD providing results from both the OrthoMCL [18] and InParanoid [19] algorithms.

Having agreed to use standardized protein sequence datasets as inputs, we next considered the existing algorithmic approaches to the determination of homology that would best meet our objectives. We chose the phylogenetic tree-based approach because it is based on an explicit evolutionary model that can be computationally evaluated. Moreover, the trees are amenable to intuitive graphical output that facilitates the rapid identification of homology sets by curators (see “*Tree-based propagation of annotations to homologous genes*” below). We are using trees generated by the PANTHER project (http://www.pantherdb.org/) based on our standardized protein-coding gene sets. The trees also include protein sequences from 34 other species to provide a more complete phylogenetic spectrum. The quality of the trees was assessed by comparing the trees to “ortholog clusters” generated by the OrthoMCL algorithm for the same protein sets. The agreement was very good overall: of the 412 OrthoMCL clusters covering the comprehensively annotated Reference Genome genes, 387 (94%) were consistent with the trees. Most of the disagreements involved a relatively distant evolutionary relationship that was difficult to resolve with certainty. Manual analysis of the trees is part of the curation process to ensure that suspicious absence of presence of proteins in the trees is supported by the genome sequence and/or the multiple sequence alignments upon which the trees are determined.

#### Selecting sets of homologous genes for annotation

**Figure 1:**
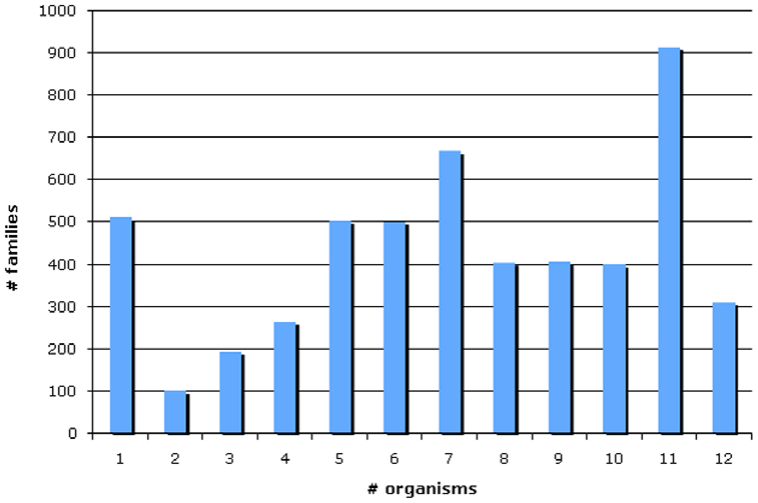
Distribution of the PANTHER families with respect to the number of reference genome species having representatives in each family.

While at present the total number of gene products in any organism is imprecisely known (largely because the full extent of post-translational modifications and alternative splicing remain uncertain) there are reasonable estimates available from the MODs for the numbers of genes encoding protein products in each genome, ranging from 4,389 in *E. coli* (data from EcoCyc Version 12.1, http://ecocyc.org) to 27,029 in *Arabidopsis thaliana* [5], for a total of roughly 200,000 genes. We are currently annotating gene families that are represented in PANTHER version 7.beta.1. Figure 1 shows how genes from the 12 GO reference genomes are distributed in these families; this reflects to some extent a bias toward coverage of human genes, which is being addressed. Nevertheless, out of 5198 families, 312 have members from all 12 reference genomes, 916 families are presents in all represented eukaryotes, and 4388 have members from at least four reference genomes. Of these 4388 families with considerable phylogenetic span, there are 3859 that already have at least one member with an experimental GO annotation from one of the MODs. These families define an initial scope for the Reference Genome project. To date, the project has annotated, in full or in part, 375 different families, slightly less than 10% of the total.

The goal of the Reference Genome project is to provide constantly up-to-date annotations for all gene families; however this work will take time. Even by initially concentrating solely on one canonical *protein* representing every gene in each genome, this strategy still presents a large and formidable target annotation list. Nevertheless, it is clear that coordination of the Reference Genome project demands a coherent prioritization of targets for curation. Accordingly, Reference Genome curators are selecting targets using the following principles:

1. Genes whose products are highly conserved during evolution, e.g. the gyrase/topoisomerase II gene family conserved from bacteria to human.
2. Genes known to be implicated in human disease and their orthologs in other taxa, e.g. the MutS homolog gene family, that includes the gene MSH6, a DNA mismatch repair protein involved in a hereditary form of colorectal cancer in humans.
3. Genes whose products are involved in known biochemical and signaling pathways, e.g. the PYGB gene (a phosphorylase) that participates in glycogen degradation.
4. Genes identified from recently published literature as having an important or new scientific impact, e.g. POU5F1 (POU class 5 homeobox 1 gene) that is important for stem cell function.

This promotes the comprehensive annotation of genes of high relevance to the current research efforts, as well as the development of the ontology to fully support those annotations.

#### Literature-based annotation

Literature curation is done by the different groups using the same method: curators read the published literature about the gene they are annotating, capturing several key pieces of information: the organism being studied, the gene product to be annotated; the type of experiment performed; the GO term(s) that best describes the gene product function/process/location; and an identifier (typically a PubMed ID) as the source for the information (citation). For each gene that is part of a curation target set, curators review existing annotations as well as add new annotations based on more recent information. If there is no literature, then the genes are immediately considered completely annotated with respect to the available experimental data. For genes with little literature, the curator reviews all available papers, but for genes for which hundreds of papers are available this is impractical. In these cases, curators assess the comprehensiveness of curation based upon recent reviews or text-mining applications, and curate key primary publications accordingly. When this is complete, the gene is considered comprehensively annotated based on the information available in the biomedical literature.

Genes that are concurrently annotated are periodically selected for annotation consistency checks among the different curation groups. Automated tests include the verification that older annotations lacking traceable evidence are replaced with annotations that adhere to the new standards, and verifying that outlier annotations, that is, those made only in one organism, are valid and not due to annotation errors. The manual review uses a peer review system in which a curator evaluates the experimentally determined annotations provided by other curators for a selected gene family. The curation consistency review process often identifies problems with the interpretation of particular GO terms. To ensure proper use of these terms in the future, they are flagged within the GO with a comment that a curator must take extra care when using these terms. For example, certain concepts, such as “development”, “differentiation” and “morphogenesis” are used with various, overlapping meanings in the literature. In GO they are distinctively defined, and we strive to ascertain that all annotations uniformly use terms as defined by the GO. The consistency review also identifies GO annotations that may be incorrect, or do not have sufficient evidence.

#### Tree-based propagation of annotations to homologous genes

The GO Reference Genome project infers functions by homology using a tree-based process that has been previously described [20]; see also ‘*Generating sets of homologous genes*’ above. The homology inference process has two steps: (1) inferring annotations of an ancestral gene, based on the (usually rather sparse) experimental annotations of its modern descendants, and (2) propagating those ancestral annotations to other descendants by inheritance. For the Reference Genome project, both of these steps are documented by an evidence trail that allows GO users to evaluate the inferences that were made. In the first step, a curator annotates an ancestral node in the phylogenetic tree, based on one or more experimentally annotated extant sequences. To document this step, a tree node (with a stable identifier) is associated with both a GO term identifier, and evidence for the association (the set of experimentally annotated sequences that descend from the annotated node). In the second step, this annotation is propagated to all its descendants (by assuming inheritance as the norm), unless the curator explicitly annotates a descendant as having lost the annotation and provides a citation for this statement. To document this step, a modern-day sequence is associated with both a GO term identifier, and evidence for the association (the annotated ancestral tree node identifier). The two documented steps allow each homology annotation to be traced through to its ancestral node (exactly what inference was made), and then to the modern-day sequences that provide experimental evidence for the annotation. This is not an automatic process, rather a curator reviews each inferred annotation with care since the function of a gene can diverge during evolution, particularly after gene duplication events that may free one of the duplicated copies from selection constraints and allow the evolution of new functionality.

**Figure 2:**
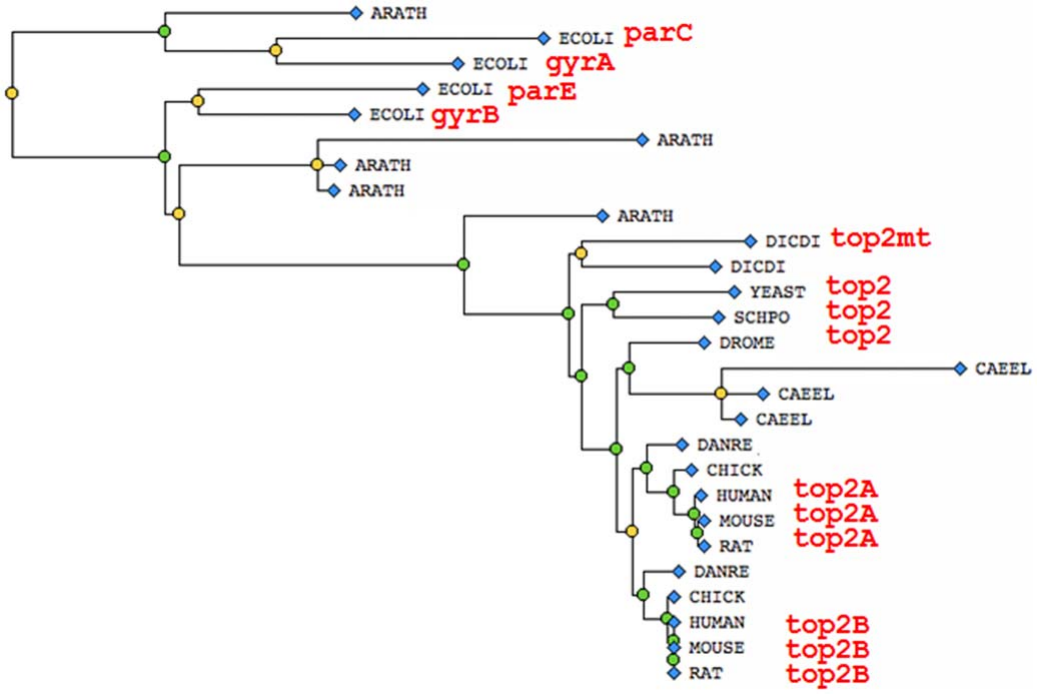
Tree representation of the TOP2 homolog set for the twelve species from the Reference Genome project. Genes having experimental data are labeled in red. Since members of all represented branches have “GO:0003918 DNA topoisomerase (ATP-hydrolyzing) activity” and a role in “GO:0007059 chromosome segregation”, the common ancestor (CA) can be inferred to also have had these functions. We thus predict that all descendents can be annotated to those terms with reasonable confidence. The sequences represented are (from top to bottom): *A. thalian*a TAIR:locus = 2075765, *E. coli* UniProt: P0AFI2 (parC), *E. coli* UniProt: P0AES4 (gyrA), *E. coli* UniProt: P20083 (parE), *E. coli* UniProt: P0AES6 (gyrB), *A. thaliana* TAIR:locus = 2146658, *A. thaliana* TAIR:locus = 2076268, *A. thaliana* TAIR:locus = 2146698, *A. thaliana* TAIR:locus = 2076201, *D. discoideum* dictyBase: DDB_G0279737 (top2mt), *D. discoideum* dictyBase: DDB_ G0270418 (top2), *S. cerevisiae* SGD:S000005032 (TOP2), *S. pombe* GeneDB SPBC1A4.03c (top2), *D. melanogaster* FlyBase FBgn0003732 (top2), *C. elegans* WormBase WBGene00019876 (R05D3.1), *C. elegans* WormBase WBGene00022854 (cin-4), *C. elegans* WormBase WBGene00021604 (Y46H3C.4), *D. reiro* ZFIN ZDB-GENE-030131-2453 (top2A), *D. reiro* ZFIN ZDB-GENE-041008-136 (top2B), *G. gallus* UniProt:O42130 (top2A), *H. sapiens* UniProt:P11288 (top2A), *M. musculus* MGI:98790 (top2A), *R. norvegius* RGD: 62048 (top2A), *G. gallus* UniProt: O42131 (top2B), *H. sapiens* UniProt:P02880 (top2B), *M. musculus* MGI:98791 (top2B), *R. norvegius* RGD: 1586156 (top2B).

An illustration of this process is shown in Figure 2. Based on the experimental annotations, the most recent common ancestor (CA) of all DNA gyrases/topoisomerases can be inferentially annotated with “DNA topoisomerase (ATP-hydrolyzing) activity” (GO:0003918) and “chromosome segregation” (GO:0007059). Perhaps most importantly, this two-step homology inference approach defines a clear methodology for propagating annotations from the twelve reference genomes to all other organisms. The annotated ancestral node defines a point in the evolutionary history at which a particular “character” (represented by a GO annotation, in this case) was acquired. A gene is assigned an annotation inferred by homology only if it descends from the annotated ancestor, a condition that can be readily determined. To enhance the utility to other genome projects, the trees annotated by the Reference Genome curators include genes from 34 other species, in addition to the twelve Reference Genomes.

## Results

### Improvements to annotations

Gene products selected for concurrent annotation in the course of the Reference Genome project have improved the breadth and depth of annotation coverage. As of November 2008, we have annotated approximately 4,000 gene products. These genes have a higher percentage of annotations derived from published experimental research. Moreover, the annotation of these genes is significantly more detailed relative to when we started this project. Initially, 34% of the 4,000 genes had annotations supported by experimental data. Now, there are 71%, a 2-fold increase; while a randomly selected sample with the same number of genes, has only 52%, a 1.5-fold increase.

We might expect the Reference Genome project to yield annotations to more specific terms. Given some specificity metric for a term, we can calculate the average specificity of terms used in annotations for Reference Genome genes and compare these against the average specificity of annotations as a whole, and observe whether there has been an overall increase in specificity. Unfortunately, there is no single perfect measure of specificity. The depth of a term in the graph structure is often a poor proxy, as this is open to ontology structure bias. In this paper we use the Shannon Information Content (IC) as a proxy for specificity of a term. The IC of a term reflects the frequency of annotations to that term (or to descendants of that term), with frequently used terms yielding a lower score than infrequently used terms. The IC is calculated as follows:
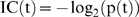
where p(t) is the probability of a gene being annotated at or below t. For example, 2.75% of genes in the GO database are currently annotated to ‘transmembrane receptor activity’, so this yields an IC of 5.18. In contrast, the more specific term GABA-B receptor activity is used for only 0.01% of genes, so this yields a higher IC of 13.29. Because annotations are propagated up the graph, the IC score must increase monotonically according to the depth in the graph – no term can have a higher IC than its descendants. But unlike the depth of the term, the IC is less open to ontology structure bias, as it is based on annotation frequency. However, the IC is subject to annotation or literature bias – if the annotated literature corpus happens to include lots of papers on transmembrane receptors, then the increased frequency of annotations will result in a lower IC. The IC is also subject to change as the annotation database changes. However, as the IC is based on the frequency rather than total number of annotations, we do not expect the IC to change radically with the annotation of new genes. We might expect a slight decrease in the IC of a term over time as annotation breadth increases, and with it the frequency of term usage.

We can measure the increase in IC on a gene set over time by measuring the average IC of the terms used to annotate the genes in that set before and after reference genome curation. Genes can have multiple annotations in each of the three branches of the GO; here we take the *maximum* IC within each branch. We then calculate the average of this maximum IC for all genes in a set to get a measure of the annotation specificity for that set. We compared this number for two sets of genes: the group of all annotated genes for all 12 gene reference genome species (which corresponds to approximately 200,000 genes), and the subset of this set corresponding to those genes that have been selected for thorough annotation. We then averaged the maximum IC values for both sets of genes before being selected for annotation by the Reference Genome project (July 2006) and again with the most recent set of annotations (December 2008). The results, shown in Table 1, are broken down by branch. For non-reference genome genes, the maximal IC has remained relatively constant or has decreased slightly. This small decrease is expected, as annotation gaps are filled in. We measured the improvement in average maximum IC of the set of reference genome annotated genes versus the baseline. As we might expect, there is an overall improvement in specificity of annotations, with annotations to biological process improving the most: the information content of the genes selected for thorough annotation has increased by about 2 for cellular component and molecular function, and by 2.44 for biological process. Since the improvement is logarithmic, an increase in 1.0 means that on average a typical gene gets annotated with a new term that is used with half the frequency of the previous most informative term.

**Table 1:** Increase in information content of the annotations of the genes from the twelve reference genomes (“All”), compared to that of the subset of genes selected for concurrent annotation (“Ref”).

		July 2006	December 2008	Change	Relative Change
Biological process	All	6.09	6.07	−0.02	+2.44
	**Ref**	**9.59**	**12.01**	**+2.42**	
Cellular component	All	4.32	4.29	−0.03	+2.06
	**Ref**	**6.43**	**8.46**	**+2.03**	
Molecular function	All	6.18	5.69	−0.49	+1.99
	**Ref**	**9.16**	**10.66**	**+1.50**	

The relative change corresponds to the sum of the changes for “All” and “Ref” sets of genes.

Another measure of the depth and breadth of GO annotations is what range of the ontology graph they cover. The graph coverage of a gene is the size of the set of terms used to annotate a gene, plus all ancestors of that term. In July 2006, the average graph coverage per reference genome gene in a reference species was 34.7, versus an average of 22.9 over all genes in all 12 species. In December 2008 this increased to 64.0 versus 27.0. This shows that the coverage of genes selected for the reference set is proportionally higher, 1.84 versus 1.18.

### Improvements to GO

The collaborative annotation of a group of similar gene products has also proven to be useful for the development of GO itself. For example, as a direct consequence of the Reference Genome project, 223 ontology changes or term modifications were made (corresponding to slightly more than 10% of the total ontology change requests during this period). Examples of requested new terms include “regulation of NAD(P)H oxidase activity”, “DNA 5′-adenosine monophosphate hydrolase activity”, “neurofilament bundle assembly”, and “quinolinate metabolic process”. We have also enhanced the ontology by adding synonyms (for example, “Y-form DNA binding” is now a synonym of “forked DNA binding”), improving definitions, and correcting inconsistencies. Examples of terms where definitions and inconsistencies have been corrected include “electron transport” (replaced by two terms: “electron transport chain” and “oxidation reduction”), and “secretory pathway” (replaced by two terms: “exocytosis” and “vesicle-mediated transport”).

### Visualization of annotations in multiple species

**Figure 3:**
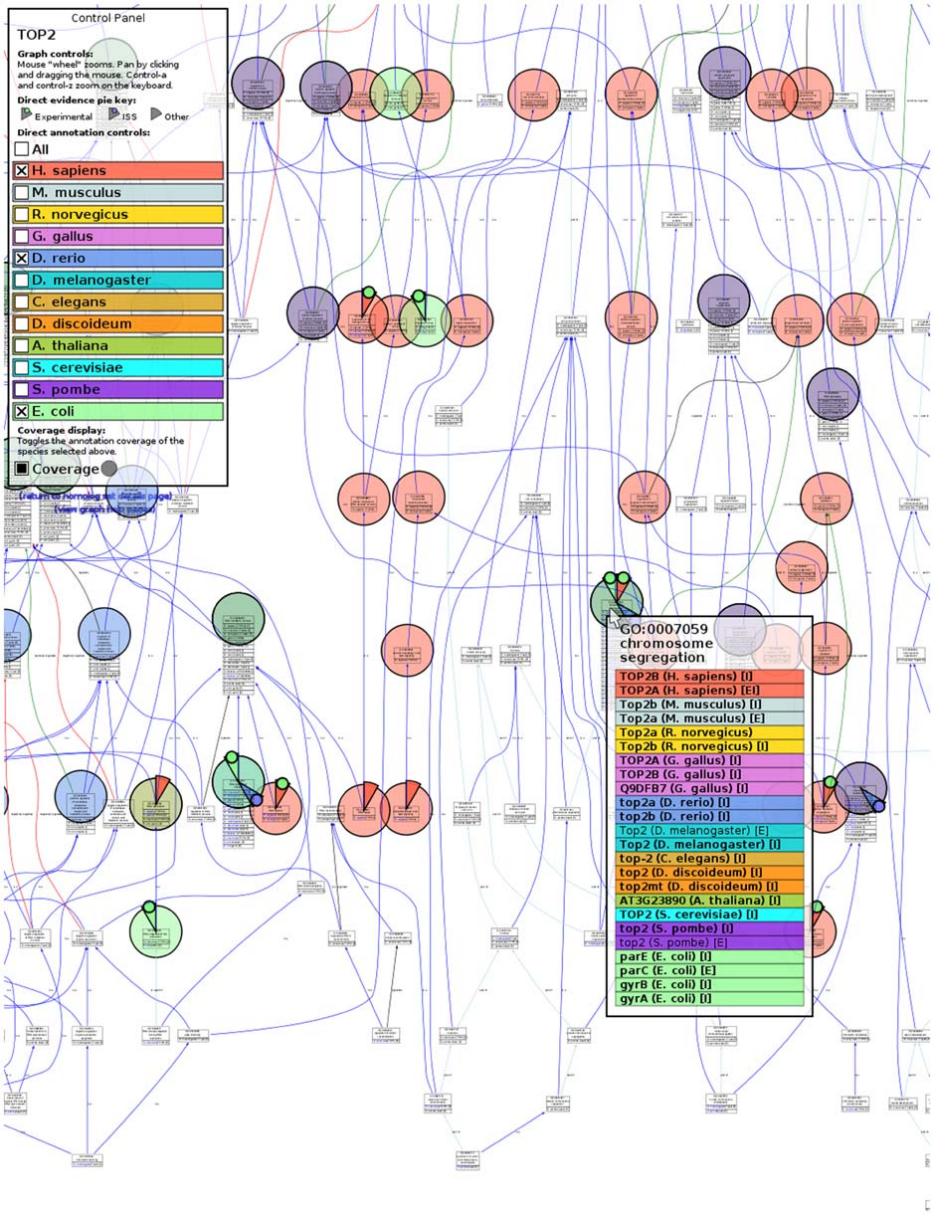
The Gene Ontology's brower AmiGO displays Comparison Graph for genes presents in homolosets. Those show all annotations, both experimental (evidence codes: IDA, IMP, IGI, IPI, IEP) as well as those inferred from sequence similarity to an experimentally characterized gene (ISS) and by curators (IC). Direct annotations to a GO term are indicated by colored wedges. Different species are represented by different colors. What species to display can be selected from the Control Panel on the righ hand side (here, the species selected are *H. sapiens*, *D. reiro*, and *E. coli*). The wedges also contain a small color-coded circle that indicates whether the annotation to a term is based on experimental data (green), supported by sequence similarity (blue), or is annotated with other evidence (no circle in the wedge). Mousing over a term leads to the display of the term ID, term name, and a complete list of annotations to that term by species. Here we show the term “chromosome segreagation”, for which five of the twelve species have experimental data to support that annotation. Annotations based on experimental data are indicated by “E”, and those based on sequence similarity by an “I”.

GO annotations may be viewed using AmiGO, the GOC browser (http://amigo.geneontology.org/) [21]. In the latest release of AmiGO a number of new displays are available that are specifically designed for public browsing of data from the Reference Genome project. For each homolog set there is a link to a “Comparison Graph” that allows the user to easily visualize the common functions for each member in gene family as well as those particular to certain organisms or groups of organisms as shown in Figure 3.

## Discussion

The aim of the Reference Genome project is to provide a source of comprehensive and reliable GO annotations for twelve key genomes based upon rigorous standards. This endeavor faces many difficult challenges, such as: the determination and provision of reference protein sets for each genome; the establishment of gene families for curation; the application of consistent best practices for annotation; and the development of methodologies for evaluating progress towards our goal. Although this is a laborious effort, steady progress is being made in developing this resource for the research community. This initiative has propelled the GOC into the provision of standardized protein sets for these genomes that we expect to be of broad utility beyond the Reference Genome project. By engaging curators from across the MODs in joint discussions we are observing improvements in curation consistency and refinement of the GOC best practices guidelines (see http://geneontology.org/GO.annotation.conventions.shtml). The genes that have been targeted by the Reference Genome project have significantly improved annotation specificity as compared to their previous annotations, and the number of genes annotated by inference through homology has also increased. This increased breadth and depth of genome coverage in the annotations is one of the major goals of the project. An additional benefit has been the improvements to the GO itself, and this will consequently improve the accuracy of inferences based on these annotations. Genomes that are fully and reliably functionally annotated empower scientific research, as they are essential for use in the analysis of many high-throughput methodologies and for the automated inferential annotation of other genomes, a major motivation of the Reference Genome project's work. We encourage users to communicate with the GO Consortium (send e-mail to gohelp@geneontology.org) with questions or suggestions for improvements to better achieve this aim.

### Data availability

Access to all GOC software and data is free and without constraints of any kind. An overview of the project as well as links to all resources described below can be found at http://geneontology.org/GO.refgenome.shtml. Annotations made by the databases participating in the Reference Genome project are available from the GOC website in gene_association file format (http://geneontology.org/GO.current.annotations.shtml). The protein sequence datasets are available for the community as a standardized resource from http://geneontology.org/gp2protein/, and as FASTA sequence files here: ftp://ftp.pantherdb.org/genome/pthr7.0. These sets provide a representative protein sequence for each protein-coding gene in each genome, cross-referenced to UniProt whenever possible, but augmented with RefSeq and Ensembl protein identifiers as well. The exact queries used to gather statistics for the annotation improvement reports can be found at: http://geneontology.org/GO.database.schema-with-views.shtml.

## References

[1] Bourne PE, McEntyre J (2006). Biocurators: contributors to the world of science.. PLoS Comput Biol.

[2] Howe D, Costanzo M, Fey P, Gojobori T, Hannick L (2008). Big data: the future of biocuration.. Nature.

[3] The Gene Ontology Consortium (2000). Gene ontology: tool for the unification of biology.. Nat Genet.

[4] The Gene Ontology Consortium (2008). The Gene Ontology project in 2008.. Nucleic Acids Res.

[5] Rhee SY, Wood V, Dolinski K, Draghici S (2008). Use and misuse of the gene ontology annotations.. Nat Rev Genet.

[6] Camon EB, Barrell DG, Dimmer EC, Lee V, Magrane M (2005). An evaluation of GO annotation retrieval for BioCreAtIvE and GOA.. BMC Bioinformatics.

[7] Dolan ME, Ni L, Camon E, Blake JA (2005). A procedure for assessing GO annotation consistency.. Bioinformatics.

[8] Artamonova II, Frishman G, Gelfand MS, Frishman D (2005). Mining sequence annotation databanks for association patterns.. Bioinformatics.

[9] Iliopoulos I, Tsoka S, Andrade MA, Enright AJ, Carroll M (2003). Evaluation of annotation strategies using an entire genome sequence.. Bioinformatics.

[10] Smith RF (1996). Perspectives: sequence data base searching in the era of large-scale genomic sequencing.. Genome Res.

[11] Smith TF, Zhang X (1997). The challenges of genome sequence annotation or “the devil is in the details”.. Nat Biotechnol.

[12] Alexeyenko A, Linberg J, Perz-Bercoff A, Sonnhammer ELL (2006). Overview and comparison of ortholog databases.. Dru Discovery Today: Technologies.

[13] Dolinski K, Botstein D (2007). Orthology and functional conservation in eukaryotes.. Annu Rev Genet.

[14] Penkett CJ, Morris JA, Wood V, Bahler J (2006). YOGY: a web-based, integrated database to retrieve protein orthologs and associated Gene Ontology terms.. Nucleic Acids Res.

[15] Heinicke S, Livstone MS, Lu C, Oughtred R, Kang F (2007). The Princeton Protein Orthology Database (P-POD): a comparative genomics analysis tool for biologists.. PLoS ONE.

[16] Mi H, Guo N, Kejariwal A, Thomas PD (2007). PANTHER version 6: protein sequence and function evolution data with expanded representation of biological pathways.. Nucleic Acids Res.

[17] Thomas PD, Campbell MJ, Kejariwal A, Mi H, Karlak B (2003). PANTHER: a library of protein families and subfamilies indexed by function.. Genome Res.

[18] Li L, Stoeckert CJ, Roos DS (2003). OrthoMCL: identification of ortholog groups for eukaryotic genomes.. Genome Res.

[19] Berglund AC, Sjolund E, Ostlund G, Sonnhammer EL (2008). InParanoid 6: eukaryotic ortholog clusters with inparalogs.. Nucleic Acids Res.

[20] Thomas PD, Mi H, Lewis S (2007). Ontology annotation: mapping genomic regions to biological function.. Curr Opin Chem Biol.

[21] Carbon S, Ireland A, Mungall CJ, Shu S, Marshall B (2009). AmiGO: online access to ontology and annotation data.. Bioinformatics.

